# Symptom Diaries of Patients with Midgut Neuroendocrine Tumors Treated with ^177^Lu-DOTATATE

**DOI:** 10.2967/jnumed.120.258897

**Published:** 2021-12

**Authors:** Jonathan R. Strosberg, Rajaventhan Srirajaskanthan, Ghassan El-Haddad, Edward M. Wolin, Beth A. Chasen, Matthew H. Kulke, David L. Bushnell, Martyn E. Caplin, Richard P. Baum, Andrew E. Hendifar, Kjell Öberg, Philippe Ruszniewski, Paola Santoro, Per Broberg, Oscar R. Leeuwenkamp, Eric P. Krenning, for the NETTER-1 study group

**Affiliations:** 1 H. Lee Moffitt Cancer Center and Research Institute, Tampa, Florida;; 2 Neuroendocrine Tumour Unit, Department of Gastroenterology, King’s College Hospital, London, United Kingdom;; 3 Center for Carcinoid and Neuroendocrine Tumors, Tish Cancer Institute at Mount Sinai, New York, New York;; 4 Department of Nuclear Medicine, Division of Diagnostic Imaging, M.D. Anderson Cancer Center, University of Texas, Houston, Texas;; 5 Section of Hematology and Medical Oncology, Boston University and Boston Medical Center, Boston, Massachusetts;; 6 Department of Radiology, University of Iowa, Iowa City, Iowa;; 7 Neuroendocrine Tumour Unit, Royal Free Hospital, London, United Kingdom;; 8 Curanosticum Wiesbaden-Frankfurt, DKD Helios Clinic, Wiesbaden, Germany;; 9 Samuel Oschin Comprehensive Cancer Institute, Cedars-Sinai Medical Center, Los Angeles, California;; 10 Department of Medical Sciences, Uppsala University, Uppsala, Sweden;; 11 Division of Gastroenterology and Pancreatology, Hôpital Beaujon, Clichy, France;; 12 Advanced Accelerator Applications, Millburn, New Jersey;; 13 Advanced Accelerator Applications, Geneva, Switzerland; and; 14 Cyclotron Rotterdam BV, Erasmus University Medical Center, Rotterdam, The Netherlands

**Keywords:** ^177^Lu, neuroendocrine, symptom diary, NETTER-1

## Abstract

We report the impact of ^177^Lu-DOTATATE treatment on abdominal pain, diarrhea, and flushing, symptoms that patients with advanced midgut neuroendocrine tumors often find burdensome. **Methods:** All patients enrolled in the international randomized phase 3 Neuroendocrine Tumors Therapy (NETTER-1) trial (^177^Lu-DOTATATE plus standard-dose octreotide long-acting repeatable [LAR], *n* = 117; high-dose octreotide LAR, *n* = 114) were asked to record the occurrence of predefined symptoms in a daily diary. Change from baseline in symptom scores (mean number of days with a symptom) was analyzed using a mixed model for repeated measures. **Results:** Patients (intent-to-treat) who received ^177^Lu-DOTATATE experienced a significantly greater decline from baseline in symptom scores than patients who received high-dose octreotide LAR. For ^177^Lu-DOTATATE, the mean decline in days with abdominal pain, diarrhea, and flushing was 4.10, 4.55, and 4.52 d per 4 wk, respectively, compared with 0.99, 1.44, and 2.54 d for high-dose octreotide LAR. The mean differences were 3.11 d (95% CI, 1.35–4.88; *P* = 0.0007) for abdominal pain, 3.11 d (1.18–5.04; *P* = 0.0017) for diarrhea, and 1.98 d (0.08–3.88; *P* = 0.0413) for flushing, favoring ^177^Lu-DOTATATE. A positive repeated-measures correlation was found between diary-recorded symptom scores and questionnaire-recorded pain, diarrhea, and flushing. **Conclusion:** In addition to efficacy and quality-of-life benefits, symptom diaries from NETTER-1 demonstrated that treatment with ^177^Lu-DOTATATE was associated with statistically significant reductions in abdominal pain, diarrhea, and flushing, constituting the core symptoms of patients with progressive midgut neuroendocrine tumors, compared with high-dose octreotide LAR, supporting a beneficial effect of ^177^Lu-DOTATATE on health-related quality of life.

## Visual Abstract

**Figure:**
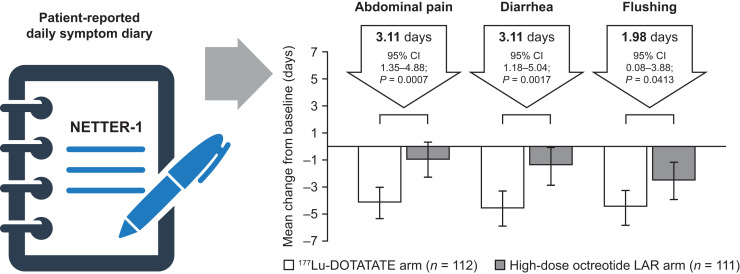

Neuroendocrine tumors (NETs) of the midgut are the most common type of gastrointestinal NET, affecting approximately 0.63–1.65 patients per 100,000 standard population per year, and are associated with 5-y survival rates of less than 50% in patients with metastatic disease (*1*–*3*). Patients with advanced midgut NETs frequently develop symptoms due to tumor growth or hormone secretion (*4*). Carcinoid syndrome, the hormonal disorder most closely associated with midgut NETs, consists primarily of diarrhea and flushing (*5*). Carcinoid syndrome has been found to be present in 32%–72% of patients with midgut NETs (*6*,*7*).

Carcinoid syndrome is caused by the secretion of serotonin and other vasoactive substances (*5*,*8*). Patients with chronic elevations of blood serotonin may also develop carcinoid heart disease, which ultimately leads to right ventricular dysfunction and to substantial morbidity and mortality (*9*–*11*). Abdominal pain is another symptom frequently associated with midgut NETs and is most commonly related to tumor volume and tumor-associated desmoplasia but can also develop as a result of hormone secretion (*12*,*13*).

Patient-reported outcomes (PROs) such as experience of symptoms, self-assessments of health or treatment, or formal health-related quality-of-life (HRQoL) questionnaires are important instruments to inform clinicians about the effects of treatment on the patient’s health status as a composite of beneficial treatment effect and side effects (*13*). PROs in a randomized controlled trial setting are particularly useful to evaluate the impact of a study drug on the patient’s health status and are becoming more important from a regulatory point of view as they capture the patient perspective regarding the disease, burden of the disease, and impact of treatments. To date, few clinical studies in NETs have reported a HRQoL benefit (*14*–*16*), highlighting the general difficulty in demonstrating a positive PRO measures outcome in cancer trials (*17*). Disease progression in patients with NETs is associated with a deterioration in HRQoL, due both to tumor growth and to progressive carcinoid syndrome (*18*).

Somatostatin analogs are typically used as first-line systemic therapy for NETs, and the clinical benefit of symptomatic relief they provide has also been supported by improved HRQoL (*19*). Telotristat ethyl has been shown to improve symptoms of diarrhea associated with carcinoid syndrome, although the effects on tumor growth are unknown (20). In the RADIANT 4 study, treatment with everolimus prolonged progression-free survival (PFS) versus placebo (both with supportive care) in patients with advanced nonfunctional gastrointestinal and lung NETs (*3*).

The Neuroendocrine Tumors Therapy (NETTER-1) trial was an international phase 3 study that randomized patients with progressive midgut NETs to receive either high-dose (60 mg) octreotide long-acting repeatable (LAR) alone (control arm) or peptide receptor radionuclide therapy (PRRT) with the radiolabeled somatostatin analog ^177^Lu-DOTATATE plus best supportive care with standard-dose (30 mg) octreotide LAR (investigational arm) (*21*). The trial met its primary endpoint of an improvement in PFS compared with high-dose octreotide LAR. The study included 2 separate PRO assessments: HRQoL questionnaires and patient symptom diaries. HRQoL was assessed using the European Organisation for Research and Treatment of Cancer (EORTC) Core Quality-of-Life Questionnaire (QLQ-C30) as well as the EORTC Gastrointestinal NET Quality-of-Life Questionnaire (QLQ-GI.NET-21), specifically developed for patients with gastrointestinal NETs. Patients in the ^177^Lu-DOTATATE treatment group experienced a statistically significant delay in time to deterioration in several HRQoL domains: global health status (hazard ratio [HR], 0.406; *P* < 0.001), physical functioning (HR, 0.518; *P* = 0.015), role functioning (HR, 0.580; *P* = 0.030), diarrhea (HR, 0.473; *P* = 0.011), fatigue (HR, 0.621; *P* = 0.030), and pain (HR, 0.566; *P* = 0.025) compared with patients in the high-dose octreotide LAR group (*15*).

Patients taking part in the NETTER-1 trial also completed daily symptom dairies. Symptom diaries are an important and distinct method for assessing the impact of treatment on symptomatology and patient outcomes. By reporting the presence or absence of a symptom from a given list of 18 symptoms, the patient diary used in the NETTER-1 study provides a means to capture symptoms on a daily basis, helping to map symptomatology experienced by the patient to HRQoL recorded on a less frequent basis. The results of these diaries are reported here, focusing on the impact of ^177^Lu-DOTATATE on diary-based assessments of abdominal pain, flushing, and diarrhea, which are the 3 symptoms regarded as most clinically relevant for patients with midgut NETs.

## MATERIALS AND METHODS

### NETTER-1 Trial Design and Patients

**FIGURE 1.**
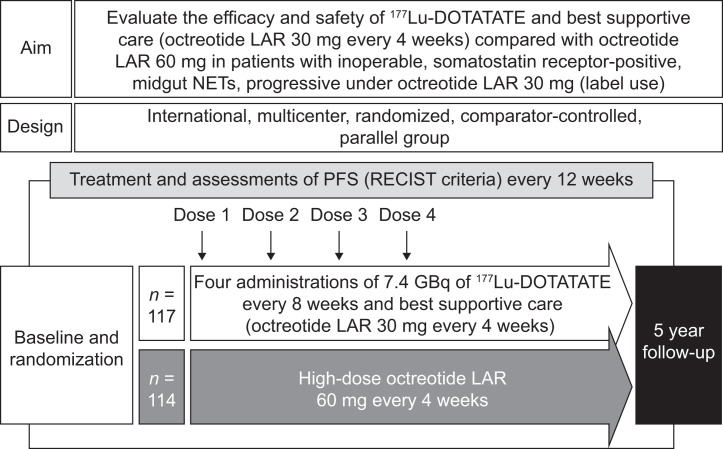
NETTER-1 trial design.

The design of the NETTER-1 trial is summarized in Figure 1. The international multicenter phase 3 NETTER-1 trial (ClinicalTrials.gov identifier NCT01578239) was a prospective randomized, controlled trial to evaluate the efficacy and safety of ^177^Lu-DOTATATE in patients with locally advanced or metastatic somatostatin receptor-positive midgut NETs with disease progression during treatment with octreotide LAR. In total, 231 adult (≥18 y old) patients were randomly assigned in a 1:1 ratio to receive either ^177^Lu-DOTATATE (consisting of 4 intravenous infusions at a dose of 7.4 GBq [200 mCi] every 8 wk plus best supportive care, consisting of intramuscular octreotide LAR 30 mg every 4 wk for symptom control; 117 patients) or high-dose intramuscular octreotide LAR (60 mg every 4 wk) alone (114 patients) (*21*).

The trial was performed in accordance with the principles of the Declaration of Helsinki, the International Conference on Harmonisation of Good-Clinical-Practice Guidelines, and all applicable regulatory requirements. The trial protocol was reviewed and approved by a review board or ethics committee at each participating center, and all participants were required to give their written informed consent before study enrollment (*21*).

Treatment continued until central confirmation of disease progression, intolerable adverse events, or withdrawal of consent. The primary endpoint was PFS, defined as the time from randomization to documented disease progression (based on independent radiologists’ review) or death from any cause. The patient-reported impact of treatment on HRQoL was a key secondary study endpoint and was assessed using EORTC QLQ-C30 and EORTC QLQ-GI.NET-21 every 12 ± 1 wk. The disease-specific QLQ-GI.NET-21 was devised and validated to supplement the generic QLQ-C30 and to include HRQoL issues tailored specifically to patients with gastrointestinal NETs (*14*). Per protocol, patients were required to complete the questionnaires until progression or until a maximum of 72 wk from random assignment had elapsed (*21*).

### Symptom Diary Assessment

**FIGURE 2.**
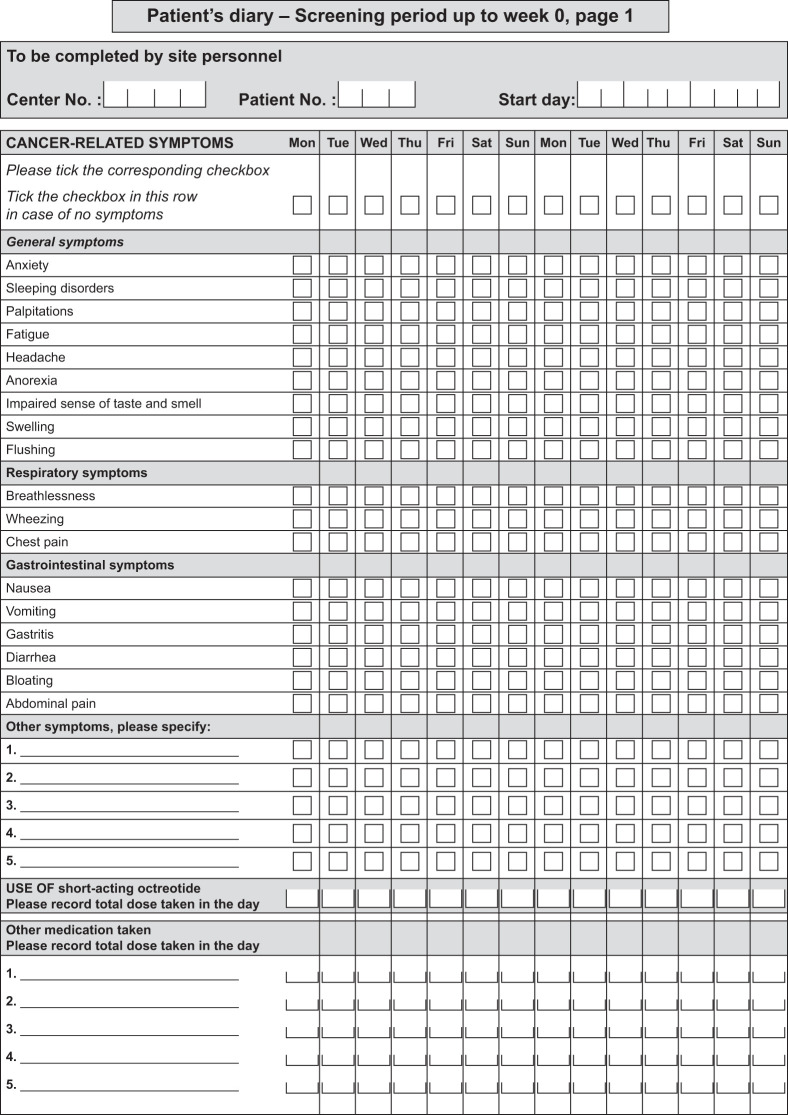
Patient diary for cancer-related symptoms. Diary contains 2 pages for each period of 4 wk, except 3 pages for screening period of up to 6 wk. At each study day, patient was asked to mark tick boxes corresponding to symptoms presented during that day and return diary at next study visit.

Per protocol, all patients enrolled in the NETTER-1 trial were asked to record the presence or absence (in the preceding 24 h) of 18 predefined symptoms by ticking boxes in a paper-based daily diary (Fig. 2). The predefined symptoms in the patient diary were subdivided into 3 categories: general symptoms (anxiety, sleeping disorders, palpitations, fatigue, headache, anorexia, impaired sense of taste and smell, swelling, and flushing), respiratory symptoms (breathlessness, wheezing, and chest pain), and gastrointestinal symptoms (nausea, vomiting, gastritis, diarrhea, bloating, and abdominal pain). Patients also had the option to include other symptoms. The current analysis focuses on the 3 symptoms most clinically relevant to patients with midgut NETs, namely abdominal pain, diarrhea, and flushing (*12,13,18*).

At each clinic visit, patients returned their diaries corresponding to the period since their previous visit. Per protocol, patients had to complete the diary up to week 72. For each symptom, the number of days on which patients reported the symptom as being present was determined as follows: at baseline, the number of days with symptoms was determined using diary data from the screening period in the 6 wk before randomization; during the randomized treatment phase (and for up to 48 wk), the number of days with symptoms was determined using diary data from each of the intervals between consecutive clinic visits.

### Statistics

A symptom score was defined as the number of days with each symptom within the 4-wk periods between clinic visits during treatment. To make the patients comparable, data from visits that were delayed by more than 1 wk were excluded and considered missing (i.e., a filter was applied that omitted observations if the time since last visit [or consent] was more than 1 wk longer than planned). Analysis was done using a mixed-model-for-repeated-measures approach, which implicitly imputes missing data under a missing-at-random assumption.

The change from baseline in the mean number of days with symptoms was assessed using a mixed model for repeated measures, adjusting for baseline symptom status, time from randomization, treatment, and interaction between time from randomization and treatment. From the model, least-squares means with 95% CIs, change from baseline, and corresponding *P* values were calculated. Analysis was performed for patients both with and without imputations.

Pearson and repeated-measures correlation analyses were performed to assess the association between the time courses of symptom scores (number of days with symptoms) and the corresponding domains in the EORTC QLQ-C30 or QLQ-GI.NET-21 (measured on a scale of 0–100 points). A positive score (>0 to 1) indicated a positive Pearson correlation, and a negative score (−1 to <0) indicated a negative Pearson correlation.

## RESULTS

### Study Population

The analysis cutoff date was June 30, 2016. In total, 231 patients were randomly assigned to treatment in the study, of whom 117 were in the ^177^Lu-DOTATATE arm and 114 in the high-dose octreotide LAR arm. The analysis was performed on all randomly assigned patients according to intent-to-treat principles.

### Symptom Diary Results

**FIGURE 3.**
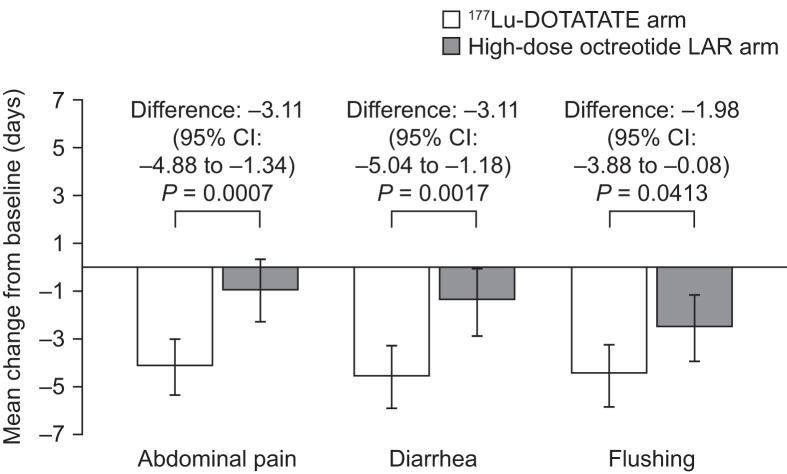
Change in mean number of days with symptoms. Negative score signifies improvement. Shown are mean change from baseline in number of days with symptoms in ^177^Lu-DOTATATE and high-dose octreotide LAR arms, adjusted for baseline symptom status. Error bars show 95% CIs. *n* = 87 (^177^Lu-DOTATATE) and *n* = 84 (high-dose octreotide LAR) patients at baseline. Number of patients with diary data decreased throughout study. Analyses were performed on intent-to-treat population.

The change from baseline in the mean number of days with symptoms for both treatment groups during each 4-wk period of the 48 wk after randomized treatment is illustrated in Figure 3.

Patients in the ^177^Lu-DOTATATE study arm experienced a significantly greater reduction from baseline in the mean number of days with symptoms (symptom score) than patients in the high-dose octreotide LAR arm, after adjustment for baseline status in the performed mixed-model-for-repeated-measures analysis.

**FIGURE 4.**
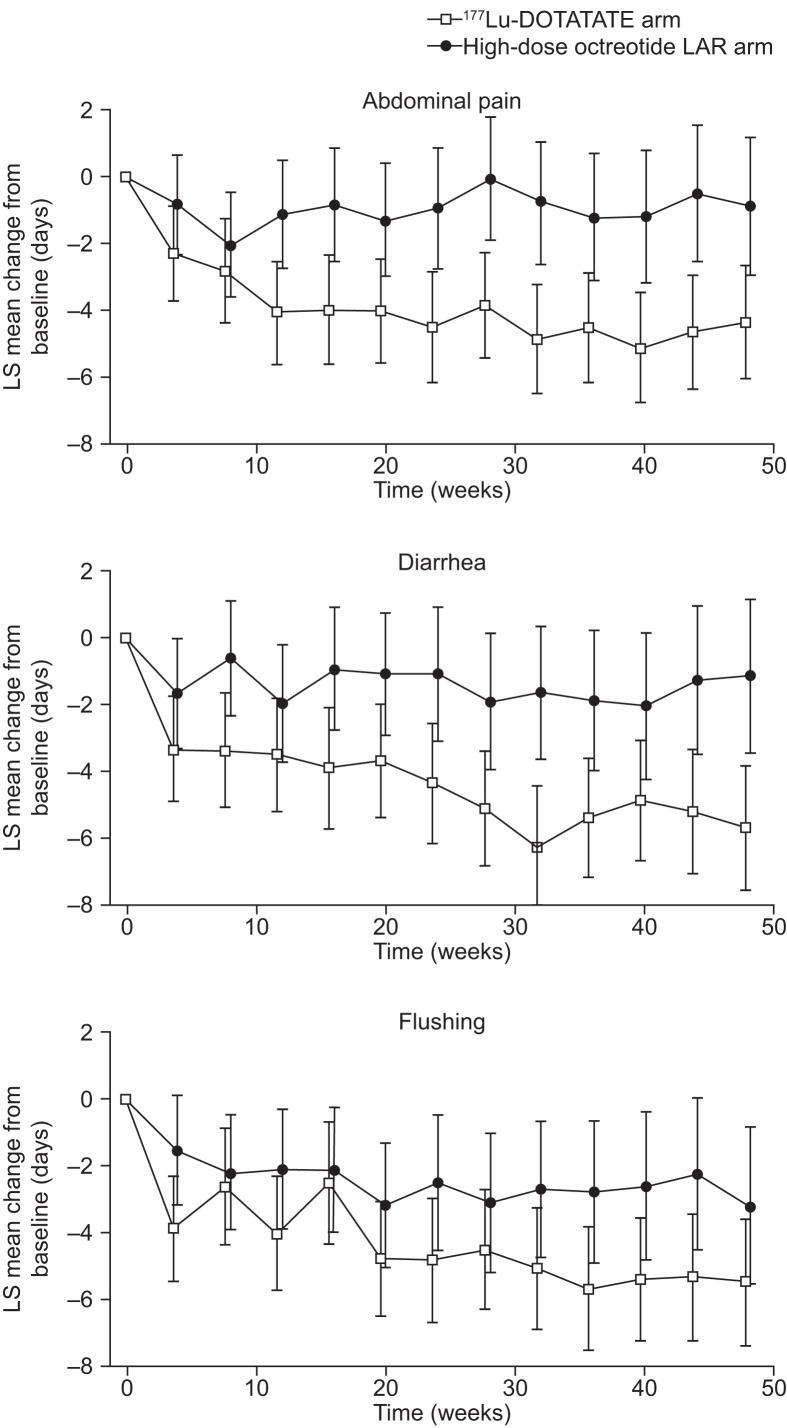
Change in mean number of days with symptoms during first 48 wk of randomized treatment phase. Negative score signifies improvement. Shown are estimated mean decline from baseline in number of days with symptoms at each time point. Estimates are least-squares (LS) means from mixed model for repeated measures. Error bars show 95% CIs. Analyses were performed on intent-to-treat population. There were 87 ^177^Lu-DOTATATE patients and 84 high-dose octreotide LAR patients at baseline. Number of patients with diary data decreased throughout study.

The estimated mean reduction from baseline in the number of days with symptoms at each time point during the randomized treatment phase is depicted in Figure 4.

For the ^177^Lu-DOTATATE group, the mean decline in number of days with abdominal pain (over a 48-wk treatment period) was 4.10 d per 4 wk, whereas in the high-dose octreotide LAR group, the mean decline was 0.99 d. The difference between the group means was 3.11 d per 4 wk (95% CI, 1.34–4.88) favoring ^177^Lu-DOTATATE. This difference was statistically significant (*P* = 0.0007).

The mean decline in number of days with diarrhea in patients receiving ^177^Lu-DOTATATE and high-dose octreotide LAR was 4.55 and 1.44 d, respectively. The difference between the group means was 3.11 d per 4 wk (95% CI, 1.18–5.04; *P* = 0.0017) favoring ^177^Lu-DOTATATE.

The mean decline in number of days with flushing in patients receiving ^177^Lu-DOTATATE and high-dose octreotide LAR was 4.52 and 2.54 d, respectively. The difference between the group means was 1.98 d per 4 wk (95% CI, 0.08–3.88; *P* = 0.041) favoring ^177^Lu-DOTATATE.

**FIGURE 5.**
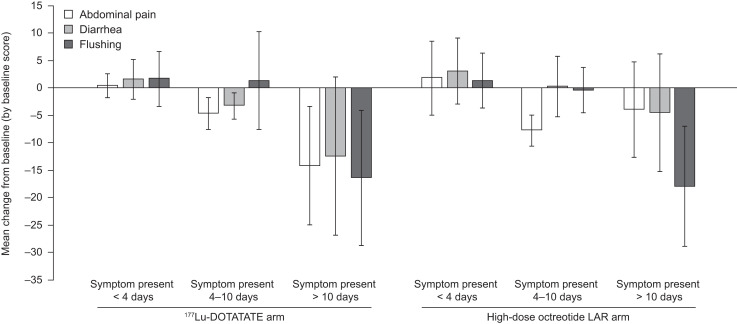
Mean change from baseline in diary symptoms over 48 wk by duration of symptoms at baseline. Negative score signifies improvement. Shown are mean changes from baseline in symptom scores in ^177^Lu-DOTATATE and high-dose octreotide LAR arms. Error bars show SD. Number of patients with diary data decreased throughout study. Analyses were performed on safety analysis set.

The treatment arms were well balanced at baseline in terms of symptom-days categories (i.e., symptoms present for <4 d, 4–10 d, or >10 d) (Table 1). Although variable, the greatest improvements in symptoms over 48 wk were generally seen in patients whose symptoms were present for more than 10 d at baseline. This was particularly the case for patients in the ^177^Lu-DOTATATE arm, with improvements compared with patients in the high-dose octreotide LAR arm observed for abdominal pain (−14.2 vs. −4.0) and diarrhea (−12.5 vs. −4.6), with flushing similar between arms (−16.4 vs. −18.0) (Fig. 5).

**TABLE 1:** Baseline Symptom Score (Safety Analysis Set)

	Baseline number of days with symptoms					
	^177^Lu-DOTATATE (*n* = 112)			High-dose octreotide LAR (*n* = 111)		
Symptom	<4 d	4–10 d	>10 d	<4 d	4–10 d	>10 d
Abdominal pain	50 (44.6)	12 (10.7)	25 (22.3)	45 (40.5)	13 (11.7)	26 (23.4)
Diarrhea	31 (27.7)	14 (12.5)	42 (37.5)	35 (31.5)	21 (18.9)	28 (25.2)
Flushing	43 (38.4)	17 (15.2)	27 (24.1)	45 (40.5)	13 (11.7)	26 (23.4)

Data are number followed by percentage in parentheses.

### Correlation Analysis

To validate the symptom score further, a Pearson correlation analysis between diary symptom scores and HRQoL questionnaire scores was performed for the corresponding symptoms measured by the EORTC QLQ-C30 or the QLQ-GI.NET-21.

**FIGURE 6.**
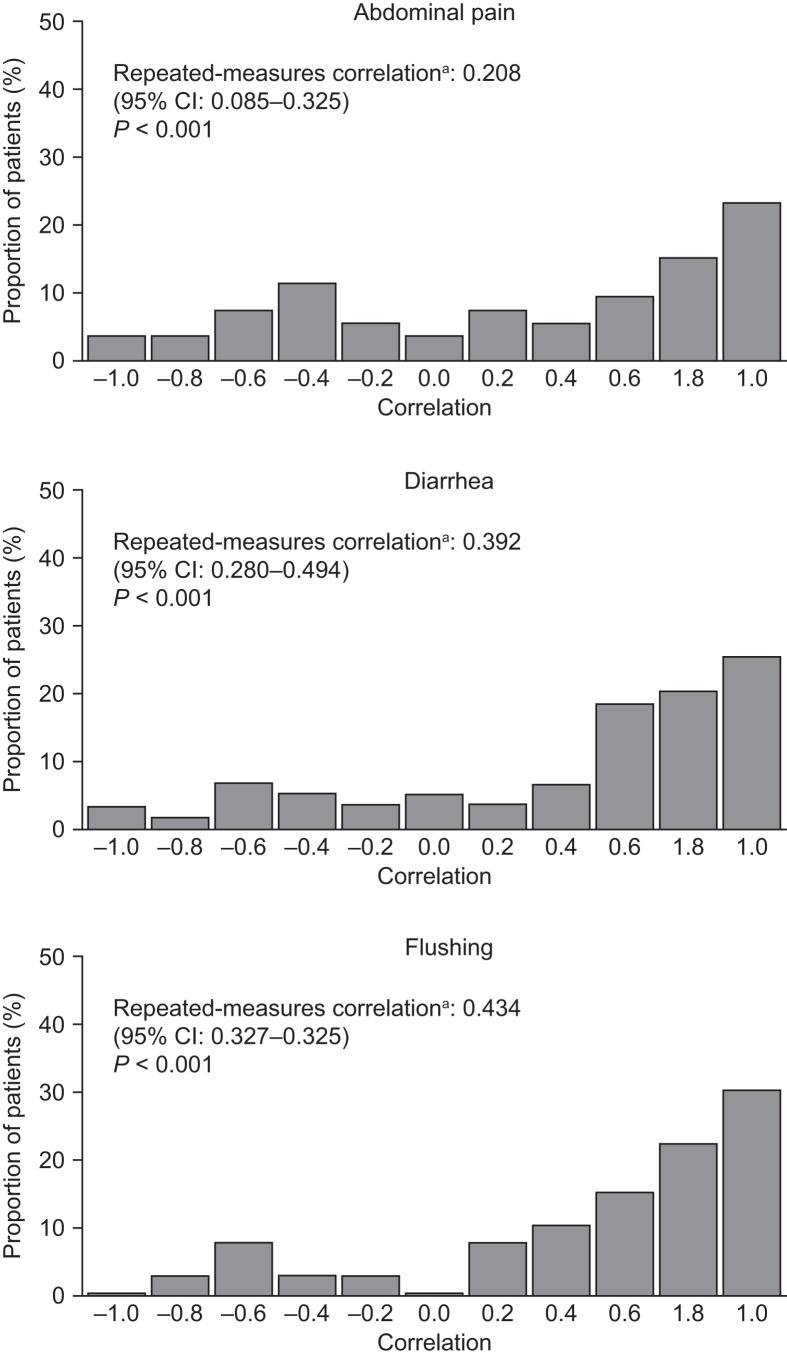
Correlation between diary-recorded and EORTC QLQ-C30– or QLQ-GI.NET-21–reported evolution of symptoms. Correlation coefficients measuring degree of association between time course of symptom score (number of days with symptoms) and quality-of-life score (0–100 on EORTC QLQ-C30 or QLQ-GI.NET-21 scale) for individual patients. Positive value (>0 to 1) indicates positive correlation, and negative value (−1 to < 0) indicates negative correlation. ^a^Summary of correlations across all patients, using Bland–Altman method (*24*).

As illustrated in Figure 6, the number of patients with a positive correlation (>0 to 1) far exceeded the number of patients with a negative correlation (−1 to <0), demonstrating a strong degree of correlation between diary-recorded symptom scores (abdominal pain, diarrhea, and flushing) and questionnaire-recorded pain and diarrhea (from the EORTC QLQ-C30) and hot flushes (from the EORTC QLQ-GI.NET-21). This finding therefore suggests face validity of the patient diary concept.

The repeated-measures correlation results were 0.39, 0.21, and 0.43 for diarrhea, pain, and flushing, respectively. All *P* values were below 0.001, signifying statistical significance and a positive Pearson correlation (with no imputations).

## DISCUSSION

Daily diaries from the phase 3 NETTER-1 trial provided information on the presence or absence of individual symptoms such as abdominal pain, diarrhea, and flushing, the 3 symptoms most commonly associated with and relevant to patients with midgut NETs.

Analysis of these patient-reported daily symptom diaries from NETTER-1 demonstrated that treatment with ^177^Lu-DOTATATE was associated with statistically significant reductions from baseline in the mean number of days with all 3 symptoms (abdominal pain, diarrhea, flushing) compared with high-dose octreotide LAR treatment.

These findings add important new HRQoL results, namely significant improvements in symptoms, to the previously reported results from NETTER-1 of a statistically significant delay in the decline of HRQoL (including global health status, physical functioning, role functioning, diarrhea, fatigue, and pain domains) for patients in the ^177^Lu-DOTATATE treatment arm compared with those in the high-dose octreotide LAR arm. It is noteworthy that there was a statistically significantly longer time to deterioration from baseline in HRQoL in the ^177^Lu-DOTATATE arm than in the control arm for the domains of diarrhea (HR, 0.47; *P* = 0.011) and abdominal pain (HR, 0.57; *P* = 0.025) (*15*).

One difference between this study and the previously reported HRQoL analysis is that, whereas the symptom diary analysis demonstrated a statistically significant symptom improvement in flushing with ^177^Lu-DOTATATE compared with high-dose octreotide LAR, no difference in symptom improvement rates occurred between treatment arms in the HRQoL analysis (*15*). However, HRQoL instruments involve a recall period during which patients average their experience in terms of health status. The diary captures symptomatology on a daily basis, which ultimately reflects into the patient’s HRQoL. It is useful to note that the endocrine scale mixes different aspects (i.e., flushing and sweats), whereas the diary symptom score addresses only flushing.

Overall, in the present study, diary-recorded symptoms and HRQoL questionnaire symptom scores demonstrated a high degree of correlation at the individual-patient level. The analysis of symptom diaries from the NETTER-1 trial therefore provides corroboration that treatment with ^177^Lu-DOTATATE among patients with progressive midgut NETs not only is efficacious (improving PFS and overall response rate) but also can palliate clinically relevant symptoms compared with high-dose octreotide LAR. This information is particularly relevant when considering alternative systemic treatments in midgut NET patients, such as everolimus, which may exacerbate symptoms such as diarrhea.

PROs such as those recorded in the daily diaries used in NETTER-1 provide important information on the potential impact of a treatment on a patient’s self-assessed experience of symptoms, symptom burden, or quality-of-life measures and therefore represent a key patient-centered clinical trial endpoint. Although patient diaries have been used occasionally in other therapeutic areas, few studies have used them to report outcome data for patients with NETs (*22*,*23*). The present study is one of the first to have shown positive outcomes of treatment on key symptoms in patients with NETs. The improvement and maintenance of an acceptable level of symptoms are important for patients with advanced NET, who typically have long treatment durations and whose disease may have had an indolent course.

A limitation of analysis of data from the NETTER-1 trial, and particularly of the PROs, is that patients (and study staff) were unable to be masked to treatment because of the differences in treatment location and administration methods between ^177^Lu-DOTATATE, which is given intravenously, and octreotide LAR, which is given intramuscularly. It is unclear whether assignment to the investigational ^177^Lu-DOTATATE arm resulted in a biased response that could have affected patient symptom perception, along with other potential perceptions of worsening of the disease and fear of radiotherapy, among others. Another limitation concerns variations in the intervals between clinic visit cycles, which may affect the method of symptom analysis. The impact of these variations was minimized by excluding visits that were delayed by more than 1 wk and using a mixed-model-for-repeated-measures analysis, which imputes missing data under a missing-at-random approach.

## CONCLUSION

This analysis of patient symptom diaries from the phase 3 NETTER-1 trial demonstrates that, in addition to improving PFS and prolonging time to deterioration in terms of HRQoL, ^177^Lu-DOTATATE treatment is also associated with statistically significant symptom relief that, compared with high-dose octreotide LAR, may benefit the patient. A significant decline was seen in the number of days that patients experienced abdominal pain, diarrhea, and flushing. Alleviation of these typical symptoms is particularly relevant to patients with progressive midgut NETs and reflects the overall benefit conferred by ^177^Lu-DOTATATE to this patient population.

## DISCLOSURE

Research was funded by Advanced Accelerator Applications (AAA), a Novartis company. AAA develops and markets treatments for cancer. Jonathan Strosberg reports personal fees from Lexicon, Ipsen, and Novartis outside the submitted work. Rajaventhan Srirajaskanthan reports educational grants from AAA outside the submitted work. Ghassan El-Haddad reports personal fees from AAA outside the submitted work. Edward Wolin reports personal fees from Ipsen, AAA, and Progenics outside the submitted work. Beth Chasen reports personal fees from AAA outside the submitted work. Martyn Caplin reports grants and personal fees from AAA and personal fees from Novartis and Ipsen outside the submitted work. Andrew Hendifar reports personal fees from AAA and from Merck, grants from PANCAN, and grants and personal fees from Ipsen outside the submitted work. Philippe Ruszniewski reports personal fees from AAA outside the submitted work. Paola Santoro is employed by AAA. Per Broberg is employed by AAA. Oscar Leeuwenkamp is employed by AAA, owns shares in AAA, and has received personal fees from Galderma outside the submitted work. Eric Krenning reports receiving travel, accommodations, or expenses from AAA and has held patents, royalties, or other intellectual property from AAA. This work was supported by AAA. Under the direction of the authors, Dr. Martin Guppy, an employee of Oxford PharmaGenesis, provided writing assistance for this article with funding from AAA. AAA reviewed for scientific accuracy. No other potential conflict of interest relevant to this article was reported.

## Notes

KEY POINTS **QUESTION:** What is the impact of ^177^Lu-DOTATATE treatment on the burdensome symptoms of abdominal pain, diarrhea, and flushing for patients with advanced midgut NETs? **PERTINENT FINDINGS:** Patients enrolled in the international randomized phase 3 NETTER-1 trial recorded the occurrence of predefined symptoms in a daily diary. Patients treated with ^177^Lu-DOTATATE plus standard-dose octreotide LAR experienced a significantly greater decline from baseline in symptom scores than patients who received high-dose octreotide LAR. **IMPLICATIONS FOR PATIENT CARE:** Compared with high-dose octreotide LAR, treatment with ^177^Lu-DOTATATE can provide clinically and statistically significant reductions in abdominal pain, diarrhea, and flushing, which are the core symptoms of patients with progressive midgut NETs.
